# Physical education and psychosocial vulnerability in adolescent suicide-related risk: a transnational cross-sectional explainable machine-learning analysis of student health survey data

**DOI:** 10.3389/fpsyt.2026.1898064

**Published:** 2026-10-02

**Authors:** Heyi Liu, Jianbin Guo, Xinwei Ma, Cheng Shi, Xiaoming Ma, Jingwei Li

**Affiliations:** 1 Tianjin College, University of Science and Technology Beijing, Tianjin, China; 2 Quality Control Office, Kailuan Mental Health Center, Tangshan, China; 3 Department of Radiology, Suzhou Hospital, Affiliated Hospital of Medical School, Nanjing University, Suzhou, China; 4 Department of Cardiology, Suzhou BenQ Medical Center, Suzhou, China; 5 Department of Neurology, Suzhou Hospital, Affiliated Hospital of Medical School, Nanjing University, Suzhou, China; 6 Department of Neurology, Nanjing University Medical School Affiliated Nanjing Drum Tower Hospital, Nanjing, China; 7 Suzhou Key Laboratory of Integrated Stroke Prevention, Treatment and Rehabilitation, Suzhou, China; 8 Suzhou Research Centre of Medical School, Nanjing University, Suzhou, China; 9 Institute of Brain Sciences, Nanjing University, Nanjing, China; 10 Nanjing Neurology Clinic Medical Center, Nanjing, China

**Keywords:** adolescent, machine learning, mental health, physical education and training, suicide

## Abstract

**Background:**

Adolescent suicide attempt is a major public health concern linked to psychosocial distress, school environment, health-risk behaviors, and physical activity patterns. This study aimed to develop an explainable XGBoost model for adolescent suicide-attempt status and to determine whether physical inactivity and low physical education (PE) participation could serve as informative school-based indicators.

**Methods:**

Data were obtained from the Global School-based Student Health Survey conducted in Argentina in 2018, Anguilla in 2016, and Jamaica in 2017. The Argentina dataset was used for model training, while Anguilla and Jamaica were combined as an independent validation cohort. The outcome was self-reported suicide attempt during the past 12 months. Candidate predictors included demographic, anthropometric, dietary, psychosocial, violence- and injury-related, tobacco-related, sexual behavior, physical activity, and PE-related variables. An XGBoost model was developed using nested cross-validation, staged hyperparameter tuning, and Bayesian fine-tuning. Model performance was evaluated by the area under the receiver operating characteristic curve (AUC). SHAP and partial dependence plots were used for interpretation.

**Results:**

A total of 27,710 adolescents were included; 5,165 (18.6%) reported at least one suicide attempt. The final model achieved an independent validation AUC of 0.7928 (95% CI: 0.7790–0.8066). The top 18 SHAP-ranked predictors retained a cumulative 10-fold cross-validated AUC of 0.7865. Loneliness was the most influential predictor, followed by sex, insomnia, early cigarette use, PE days, physical attack, school peer kindness, and physical activity. Physical inactivity and low PE participation showed nonlinear associations with predicted suicide-attempt risk.

**Conclusions:**

Loneliness and insomnia represented the psychosocial vulnerability profile, while physical inactivity and limited PE participation emerged as potentially informative school-based indicators requiring further longitudinal investigation. These findings support risk-stratified prevention strategies integrating early psychological identification with inclusive, socially supportive physical activity and PE programs.

## 1 Introduction

Adolescent mental health has become an urgent global public health priority. Globally, approximately one in seven adolescents aged 10–19 years experiences a mental disorder, and suicide is among the leading causes of death in older adolescents and young adults. Adolescence is a developmental period characterized by rapid biological, psychological, and social changes, during which sleep patterns, peer relationships, risk-taking behaviors, and physical activity habits are established. These features make schools a critical setting for both early risk identification and preventive intervention, particularly because schools provide repeated opportunities to observe behavioral patterns and deliver low-cost, population-level health promotion strategies (1).

School-based surveillance studies have shown that adolescent suicide-related behaviors are embedded in a broader psychosocial and behavioral context. Pooled analyses of the Global School-based Student Health Survey (GSHS) have reported substantial cross-national variation in suicidal ideation and suicide attempts, with individual, interpersonal, and school-related factors contributing to risk (2). Prior GSHS-based studies have linked adolescent suicidality with loneliness, insomnia, bullying victimization, violence-related experiences, substance use, and weak social support (3–5). These findings suggest that adolescent suicide-related risk should not be interpreted as an isolated psychiatric outcome, but rather as a multidimensional vulnerability pattern involving emotional distress, social disconnection, sleep disturbance, health-risk behaviors, and school environment.

Among school-based and potentially modifiable factors, physical activity and physical education are especially relevant. Current World Health Organization guidelines recommend that children and adolescents engage in an average of at least 60 minutes per day of moderate-to-vigorous physical activity, with vigorous-intensity and muscle- and bone-strengthening activities incorporated at least three days per week (1). However, physical activity in adolescents is not only a matter of individual lifestyle choice; it is also shaped by school schedules, physical education opportunities, peer participation, and the broader social climate of the school. A multi-country GSHS analysis showed that participation in physical education classes was associated with sufficient physical activity among adolescents across 65 countries (6). Therefore, physical education may represent a practical and scalable school-based platform for improving adolescent movement behavior.

Accumulating evidence also supports the mental health relevance of physical activity and sport participation in youth. School-related physical activity interventions have been reported to improve several mental health outcomes, including anxiety, resilience, well-being, and positive mental health (7). A systematic review and meta-analysis further suggested that physical activity interventions may reduce depressive symptoms in children and adolescents (8). Organized sport participation during adolescence has also been inversely associated with symptoms of anxiety and depression (9). In relation to suicidality, a systematic review and meta-analysis found that higher physical activity levels were associated with lower suicidal ideation, although prospective and intervention evidence remains limited (10). These findings support the hypothesis that physical activity and physical education may serve not only as health behaviors, but also as school-based entry points for enhancing peer connectedness, sleep regulation, self-esteem, and psychological resilience.

Nevertheless, several important gaps remain. First, previous studies have commonly evaluated physical activity, physical education, and psychosocial ri using conventional regression-based approaches, which may not fully capture nonlinear associations or complex interactions among adolescent behaviors. Second, less is known about whether physical education- and physical activity-related variables remain informative when considered alongside stronger psychosocial predictors such as loneliness, insomnia, sex, peer support, and tobacco-related behaviors. Third, from a prevention perspective, it is important to distinguish variables that are merely predictive from those that may also be actionable in school settings. Explainable machine-learning approaches can help address these gaps by combining predictive modeling with interpretable feature attribution.

Therefore, using GSHS data from Argentina, Anguilla, and Jamaica, the present study aimed to develop and validate an explainable XGBoost model for identifying adolescent suicide-related risk. Argentina 2018 was used as the training dataset, while Anguilla 2016 and Jamaica 2017 were combined as an independent validation dataset. We used SHapley Additive exPlanations (SHAP) to identify the relative contribution of psychosocial, behavioral, and physical activity-related predictors, and partial dependence plots (PDPs) to further examine the nonlinear relationships between selected predictors and predicted risk. Particular attention was given to physical education days and physical activity as school-based, modifiable indicators with potential relevance for adolescent mental health promotion.

## 2 Methods

### 2.1 Study design and data source

The cross-sectional study used data from the Global School-based Student Health Survey (GSHS) conducted in Anguilla in 2016, Jamaica in 2017, and Argentina in 2018. The GSHS is a standardized school-based survey designed to collect information on health behaviors and protective factors among school-going adolescents. The present analysis focused on adolescent suicide attempt and related demographic, psychosocial, behavioral, dietary, and physical activity-related factors.

Because of sample-size and year, country differences considerations, the Argentina 2018 dataset was used for model development, whereas Anguilla 2016 and Jamaica 2017 datasets were reserved as an independent external validation cohort. This design was selected to evaluate model transportability across different countries and survey settings.

All analyses were based on de-identified survey data. No personal level identifiable information was used in the present study.

### 2.2 Outcome variable processing

The primary outcome was self-reported suicide attempt, defined by the variable “AttemptSuicide”. For machine-learning analysis, the outcome was converted into a binary variable, with 0 indicating at least one suicidal attempt and 1 indicating no suicide attempt. When the original response coding was 1/2, the variable was recoded to 0/1 before model development. Because the original coding assigned 0 to suicide attempt and 1 to no suicide attempt, SHAP interpretation was performed with careful consideration of class direction. For clarity, positive SHAP contributions were interpreted as increasing the probability of suicide attempt after reversing the model output interpretation.

### 2.3 Machine-learning candidate predictors

Candidate predictors were selected from variables available in the GSHS dataset and included demographic characteristics, anthropometric indicators, dietary behaviors, violence- and injury-related experiences, psychosocial factors, tobacco-related behavior, sexual behavior, and physical activity-related indicators.

Continuous variables included age, height, and weight. Categorical or ordinal variables included sex, grade, hunger experience, fruit intake, vegetable intake, carbonated drink consumption, fast food intake, being physically attacked, serious injury, loneliness, insomnia, number of close friends, age at first cigarette use, sexual intercourse, physical activity, walking or riding to school, number of physical education days, and perceived kindness or helpfulness of students.

Particular attention was given to physical activity and physical education-related variables, including “PhysicalActivity”, “Walk.Ride”, and “PEDays”, because these variables represent modifiable school-based or lifestyle-related indicators relevant to adolescent mental health promotion.

### 2.4 Model development, validation, and interpretation

An extreme gradient boosting model was developed to predict the probability of suicide attempt. The binary logistic objective function was used, and the area under the receiver operating characteristic curve was selected as the main optimization metric. Predictor variables were converted into a numeric matrix before model fitting.

Model development was performed in the Argentina 2018 training cohort. A 10-fold nested cross-validation strategy was used for internal validation and hyperparameter selection. Hyperparameters were optimized through a staged tuning procedure followed by Bayesian fine-tuning. The staged tuning process included tree-structure parameters, learning rate and number of boosting rounds, subsampling parameters, and regularization parameters. Early stopping was applied when the cross-validated AUC did not improve after 30 rounds. After hyperparameter optimization, the final XGBoost model was trained on the full Argentina 2018 training cohort.

External validation was conducted in the combined Anguilla 2016 and Jamaica 2017 validation cohort. Model discrimination was evaluated using the receiver operating characteristic curve and AUC, with 95% confidence intervals estimated using the pROC package. Predicted probabilities were also dichotomized at a threshold of 0.50 to calculate sensitivity, specificity, and F1 score.

To improve interpretability, SHapley Additive exPlanations were calculated using the final XGBoost model. Global feature importance was defined as the mean absolute SHAP value for each predictor. Predictors were ranked according to SHAP importance, and a SHAP beeswarm plot was used to visualize the magnitude and direction of feature contributions. A cumulative AUC analysis was further performed by sequentially adding predictors according to their SHAP ranking, which allowed assessment of the predictive performance retained by a compact set of top-ranked features. Local SHAP analysis was conducted for the validation-set participant with the highest predicted probability to illustrate individualized risk attribution.

Partial dependence plots were generated for the top SHAP-ranked predictors to examine the marginal relationship between each predictor and the predicted probability of suicide attempt. Particular attention was given to physical activity and physical education-related variables, including physical activity, walking or riding to school, and physical education days, because these factors may represent modifiable school-based indicators relevant to adolescent mental health promotion.

All analyses were conducted using R software. Model development and validation were performed using xgboost, caret, pROC, and rBayesianOptimization. SHAP analyses were conducted using SHAPforxgboost, and partial dependence plots were generated using the pdp package.

## 3 Results

### 3.1 Baseline demographic characteristics and clinical information

Among the 27,710 adolescents included in the analysis, 5,165 (18.6%) reported at least one suicide attempt during the previous 12 months, whereas 22,545 (81.4%) did not. Compared with adolescents without suicide attempts, those reporting suicide attempts were more likely to be female and showed higher frequencies of loneliness, insomnia, physical attack, serious injury, early cigarette use, and poorer perceived peer support (**Table 1**).

**Table 1:** Demographic characteristics and clinical information of the study patients (n=27710).

P-value	Level	Overall	0	1	p
n		27710	5165	22545	
Height (mean ± SD)		1.64 ± 0.10	1.63 ± 0.09	1.65 ± 0.10	<0.0001
Weight (mean ± SD)		50.99 ± 15.55	50.61 ± 14.41	51.53 ± 17.03	<0.0001
Sex (%)	Male	12508 (45.10)	1582 ( 30.60)	10926 ( 48.50)	<0.0001
	Female	15202 (54.90)	3583 ( 69.40)	11619 ( 51.50)	
Grade (%)	7th	3284 (11.90)	556 ( 10.80)	2728 ( 12.10)	<0.0001
	8th	5472 (19.70)	1051 ( 20.30)	4421 ( 19.60)	
	9th	6416 (23.20)	1276 ( 24.70)	5140 ( 22.80)	
	10th	6958 (25.10)	1310 ( 25.40)	5648 ( 25.10)	
	11th	5543 (20.00)	936 ( 18.10)	4607 ( 20.40)	
	12th	37 ( 0.10)	36 ( 0.70)	1 ( 0.00)	
Hungry (%)	Never	19174 (69.20)	2947 ( 57.10)	16227 ( 72.00)	<0.0001
	Rarely	5632 (20.30)	1228 ( 23.80)	4404 ( 19.50)	
	Sometimes	2476 ( 8.90)	782 ( 15.10)	1694 ( 7.50)	
	Most of the Time	321 ( 1.20)	168 ( 3.30)	153 ( 0.70)	
	Always	107 ( 0.40)	40 ( 0.80)	67 ( 0.30)	
Fruits (%)	I did not eat fruit during the past 30 days	5227 (18.90)	1220 ( 23.60)	4007 ( 17.80)	<0.0001
	Less than one time per day	8904 (32.10)	1614 ( 31.20)	7290 ( 32.30)	
	1 time per day	4067 (14.70)	755 ( 14.60)	3312 ( 14.70)	
	2 times per day	3907 (14.10)	664 ( 12.90)	3243 ( 14.40)	
	3 times per day	2891 (10.40)	427 ( 8.30)	2464 ( 10.90)	
	4 times per day	1378 ( 5.00)	214 ( 4.10)	1164 ( 5.20)	
	5 or more times per day	1336 ( 4.80)	271 ( 5.20)	1065 ( 4.70)	
Vegetables (%)	I did not eat vegetables during the past 30 days	2470 ( 8.90)	594 ( 11.50)	1876 ( 8.30)	<0.0001
	Less than one time per day	7360 (26.60)	1329 ( 25.70)	6031 ( 26.80)	
	1 time per day	6371 (23.00)	1197 ( 23.20)	5174 ( 22.90)	
	2 times per day	4834 (17.40)	848 ( 16.40)	3986 ( 17.70)	
	3 times per day	4006 (14.50)	645 ( 12.50)	3361 ( 14.90)	
	4 times per day	991 ( 3.60)	176 ( 3.40)	815 ( 3.60)	
	5 or more times per day	1678 ( 6.10)	376 ( 7.30)	1302 ( 5.80)	
CarbonatedDrinks (%)	I did not drink carbonated soft drinks during the past 30 days	5868 (21.20)	1101 ( 21.30)	4767 ( 21.10)	<0.0001
	Less than one time per day	9484 (34.20)	1535 ( 29.70)	7949 ( 35.30)	
	1 time per day	4170 (15.00)	853 ( 16.50)	3317 ( 14.70)	
	2 times per day	2803 (10.10)	544 ( 10.50)	2259 ( 10.00)	
	3 times per day	1936 ( 7.00)	359 ( 7.00)	1577 ( 7.00)	
	4 times per day	1165 ( 4.20)	209 ( 4.00)	956 ( 4.20)	
	5 or more times per day	2284 ( 8.20)	564 ( 10.90)	1720 ( 7.60)	
FastFood (%)	0 days	17219 (62.10)	3143 ( 60.90)	14076 ( 62.40)	<0.0001
	1 day	5561 (20.10)	975 ( 18.90)	4586 ( 20.30)	
	2 days	2487 ( 9.00)	453 ( 8.80)	2034 ( 9.00)	
	3 days	1097 ( 4.00)	246 ( 4.80)	851 ( 3.80)	
	4 days	456 ( 1.60)	121 ( 2.30)	335 ( 1.50)	
	5 days	271 ( 1.00)	68 ( 1.30)	203 ( 0.90)	
	6 days	115 ( 0.40)	28 ( 0.50)	87 ( 0.40)	
	7 days	504 ( 1.80)	131 ( 2.50)	373 ( 1.70)	
GotAttacked (%)	0 times	23238 (83.90)	3710 ( 71.80)	19528 ( 86.60)	<0.0001
	1 time	2447 ( 8.80)	645 ( 12.50)	1802 ( 8.00)	
	2 or 3 times	1112 ( 4.00)	396 ( 7.70)	716 ( 3.20)	
	4 or 5 times	336 ( 1.20)	149 ( 2.90)	187 ( 0.80)	
	6 or 7 times	139 ( 0.50)	60 ( 1.20)	79 ( 0.40)	
	8 or 9 times	79 ( 0.30)	37 ( 0.70)	42 ( 0.20)	
	10 or 11 times	43 ( 0.20)	20 ( 0.40)	23 ( 0.10)	
	12 or more times	316 ( 1.10)	148 ( 2.90)	168 ( 0.70)	
SeriouslyInjured (%)	0 times	18074 (65.20)	3040 ( 58.90)	15034 ( 66.70)	<0.0001
	1 time	5599 (20.20)	1079 ( 20.90)	4520 ( 20.00)	
	2 or 3 times	2762 (10.00)	657 ( 12.70)	2105 ( 9.30)	
	4 or 5 times	677 ( 2.40)	186 ( 3.60)	491 ( 2.20)	
	6 or 7 times	214 ( 0.80)	71 ( 1.40)	143 ( 0.60)	
	8 or 9 times	130 ( 0.50)	37 ( 0.70)	93 ( 0.40)	
	10 or 11 times	58 ( 0.20)	18 ( 0.30)	40 ( 0.20)	
	12 or more times	196 ( 0.70)	77 ( 1.50)	119 ( 0.50)	
Lonely (%)	Never	8457 (30.50)	707 ( 13.70)	7750 ( 34.40)	<0.0001
	Rarely	7356 (26.50)	830 ( 16.10)	6526 ( 28.90)	
	Sometimes	7479 (27.00)	1609 ( 31.20)	5870 ( 26.00)	
	Most of the time	3073 (11.10)	1256 ( 24.30)	1817 ( 8.10)	
	Always	1345 ( 4.90)	763 ( 14.80)	582 ( 2.60)	
Insomnia (%)	Never	7833 (28.30)	837 ( 16.20)	6996 ( 31.00)	<0.0001
	Rarely	8545 (30.80)	1175 ( 22.70)	7370 ( 32.70)	
	Sometimes	7905 (28.50)	1688 ( 32.70)	6217 ( 27.60)	
	Most of the time	2578 ( 9.30)	1001 ( 19.40)	1577 ( 7.00)	
	Always	849 ( 3.10)	464 ( 9.00)	385 ( 1.70)	
AttemptSuicide (%)	Yes	5165 (18.60)	5165 (100.00)	0 ( 0.00)	<0.0001
	Never	22545 (81.40)	0 ( 0.00)	22545 (100.00)	
CloseFriends (%)	0	1384 ( 5.00)	446 ( 8.60)	938 ( 4.20)	<0.0001
	1	2241 ( 8.10)	581 ( 11.20)	1660 ( 7.40)	
	2	4728 (17.10)	1039 ( 20.10)	3689 ( 16.40)	
	3 or more	19357 (69.90)	3099 ( 60.00)	16258 ( 72.10)	
FirstCigarette (%)	I have never smoked cigarettes	16595 (59.90)	2334 ( 45.20)	14261 ( 63.30)	<0.0001
	7 years old or younger	494 ( 1.80)	168 ( 3.30)	326 ( 1.40)	
	8 or 9 years old	720 ( 2.60)	230 ( 4.50)	490 ( 2.20)	
	10 or 11 years old	1478 ( 5.30)	444 ( 8.60)	1034 ( 4.60)	
	12 or 13 years old	3683 (13.30)	1017 ( 19.70)	2666 ( 11.80)	
	14 or 15 years old	3671 (13.20)	775 ( 15.00)	2896 ( 12.80)	
	16 or 17 years old	1062 ( 3.80)	195 ( 3.80)	867 ( 3.80)	
	18 years old or older	7 ( 0.00)	2 ( 0.00)	5 ( 0.00)	
SexualIntercourse (%)	Yes	12216 (44.10)	2603 ( 50.40)	9613 ( 42.60)	<0.0001
	No	15494 (55.90)	2562 ( 49.60)	12932 ( 57.40)	
PhysicalActivity (%)	0 days	4031 (14.50)	996 ( 19.30)	3035 ( 13.50)	<0.0001
	1 day	3589 (13.00)	764 ( 14.80)	2825 ( 12.50)	
	2 days	4743 (17.10)	805 ( 15.60)	3938 ( 17.50)	
	3 days	3666 (13.20)	610 ( 11.80)	3056 ( 13.60)	
	4 days	2704 ( 9.80)	412 ( 8.00)	2292 ( 10.20)	
	5 days	2733 ( 9.90)	421 ( 8.20)	2312 ( 10.30)	
	6 days	1328 ( 4.80)	158 ( 3.10)	1170 ( 5.20)	
	7 days	4916 (17.70)	999 ( 19.30)	3917 ( 17.40)	
Walk.Ride (%)	0 days	9332 (33.70)	1812 ( 35.10)	7520 ( 33.40)	<0.0001
	1 day	1751 ( 6.30)	291 ( 5.60)	1460 ( 6.50)	
	2 days	1668 ( 6.00)	299 ( 5.80)	1369 ( 6.10)	
	3 days	1354 ( 4.90)	240 ( 4.60)	1114 ( 4.90)	
	4 days	1210 ( 4.40)	219 ( 4.20)	991 ( 4.40)	
	5 days	4045 (14.60)	624 ( 12.10)	3421 ( 15.20)	
	6 days	569 ( 2.10)	107 ( 2.10)	462 ( 2.00)	
	7 days	7781 (28.10)	1573 ( 30.50)	6208 ( 27.50)	
PEDays (%)	0 days	2981 (10.80)	789 ( 15.30)	2192 ( 9.70)	<0.0001
	1 day	4929 (17.80)	1025 ( 19.80)	3904 ( 17.30)	
	2 days	10279 (37.10)	1555 ( 30.10)	8724 ( 38.70)	
	3 days	717 ( 2.60)	139 ( 2.70)	578 ( 2.60)	
	4 days	582 ( 2.10)	129 ( 2.50)	453 ( 2.00)	
	5 or more days	8222 (29.70)	1528 ( 29.60)	6694 ( 29.70)	
StudentsKind (%)	Never	1901 ( 6.90)	553 ( 10.70)	1348 ( 6.00)	<0.0001
	Rarely	5216 (18.80)	1322 ( 25.60)	3894 ( 17.30)	
	Sometimes	7178 (25.90)	1440 ( 27.90)	5738 ( 25.50)	
	Most of the time	8448 (30.50)	1230 ( 23.80)	7218 ( 32.00)	
	Always	4967 (17.90)	620 ( 12.00)	4347 ( 19.30)	

### 3.2 SHapley additive exPlanations

## 4 Discussion

In this cross-sectional analysis of Global School-based Student Health Survey data from Argentina, Anguilla, and Jamaica, we developed and externally validated an explainable XGBoost model for adolescent suicide-attempt status. Argentina 2018 was used for model training, whereas Anguilla 2016 and Jamaica 2017 were combined as an independent validation cohort. The final model showed acceptable discrimination in the validation cohort, with an AUC of 0.7928 and a 95% confidence interval of 0.7790–0.8066. Because the outcome was coded as AttemptSuicide = 0 for adolescents with suicide attempt and AttemptSuicide = 1 for adolescents without suicide attempt, SHAP results were interpreted according to their direction relative to suicide-attempt risk. Because the model was trained with suicide attempt coded as 0 and no suicide attempt coded as 1, SHAP interpretation was performed according to the direction of contribution to the model output. Features that shifted predictions toward the suicide-attempt class were interpreted as contributing to a higher predicted probability of suicide attempt, whereas features shifting predictions toward the non-attempt class were interpreted as contributing to a lower predicted probability of suicide attempt.

The main finding of this study is that adolescent suicide-attempt status was not determined by a single variable, but by an interconnected psychosocial, behavioral, and school-environmental profile. The most important predictors included loneliness, sex, insomnia, early cigarette use, physical education participation, physical attack, school peer kindness, physical activity, hunger, age, close friends, injury-related variables, and several dietary or anthropometric indicators. This pattern is consistent with contemporary evidence that adolescent suicidality is shaped by multiple domains, including social isolation, sleep disturbance, sex-related vulnerability, health-risk behaviors, interpersonal violence, and social connectedness (1, 8, 11–13).

The baseline characteristics supported this multidimensional interpretation. Among 27,710 adolescents, 5,165 students, accounting for 18.6% of the analytic sample, belonged to the suicide-attempt group. Compared with adolescents without suicide attempt, those with suicide attempt were more likely to be female, report frequent loneliness and insomnia, have fewer close friends, experience physical attack or serious injury, report earlier cigarette use, and perceive a less supportive school peer environment. The contrast was particularly pronounced for loneliness and insomnia. Feeling lonely “most of the time” or “always” was reported by 39.1% of adolescents with suicide attempt, compared with 10.7% of those without suicide attempt. Similarly, insomnia-related worry “most of the time” or “always” was reported by 28.4% of adolescents with suicide attempt, compared with 8.7% of those without suicide attempt. These results are consistent with meta-analytic evidence linking loneliness and sleep disturbance to suicidal ideation and suicidal behavior (14).

The global SHAP beeswarm plot (**Figure 2**) provides a visual summary of this risk structure. Loneliness was the most influential predictor, with a mean absolute SHAP value of 0.513, followed by sex, insomnia, early cigarette use, physical education days, physical attack, school peer kindness, and physical activity. This ranking is clinically meaningful because it indicates that suicide-attempt prediction was not simply a reflection of demographic characteristics or isolated lifestyle behaviors. Instead, the model captured a broader vulnerability profile involving emotional distress, social disconnection, sleep disturbance, early health-risk behavior, adverse experiences, school climate, and movement-related factors. The prominent role of sex is also consistent with meta-analytic evidence showing that females have higher risk of suicide attempt during adolescence and young adulthood, although males have higher risk of suicide death (13).

**Figure 1:**
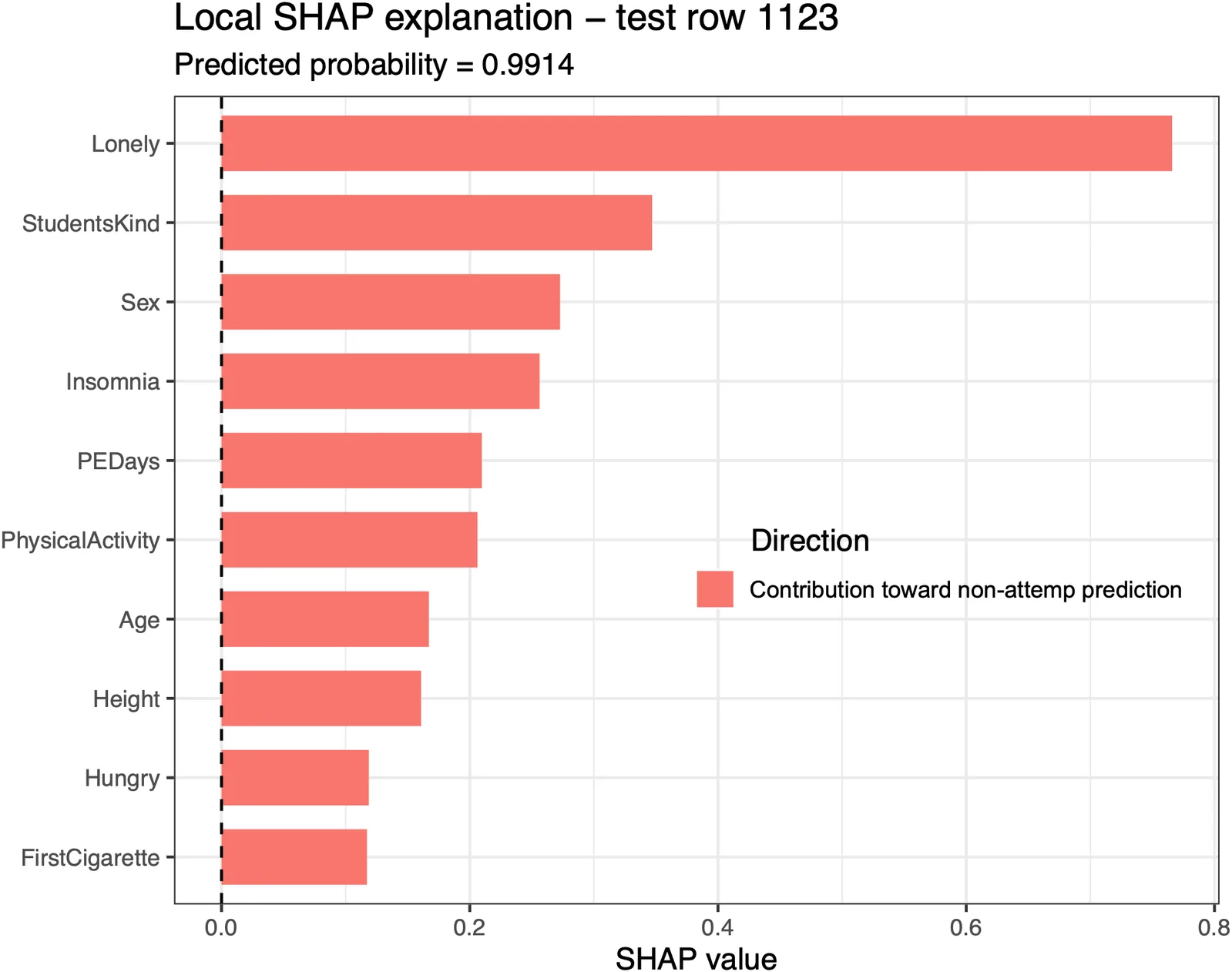
Local SHAP explanation of test row prediction in the independent validation cohort.

**Figure 2:**
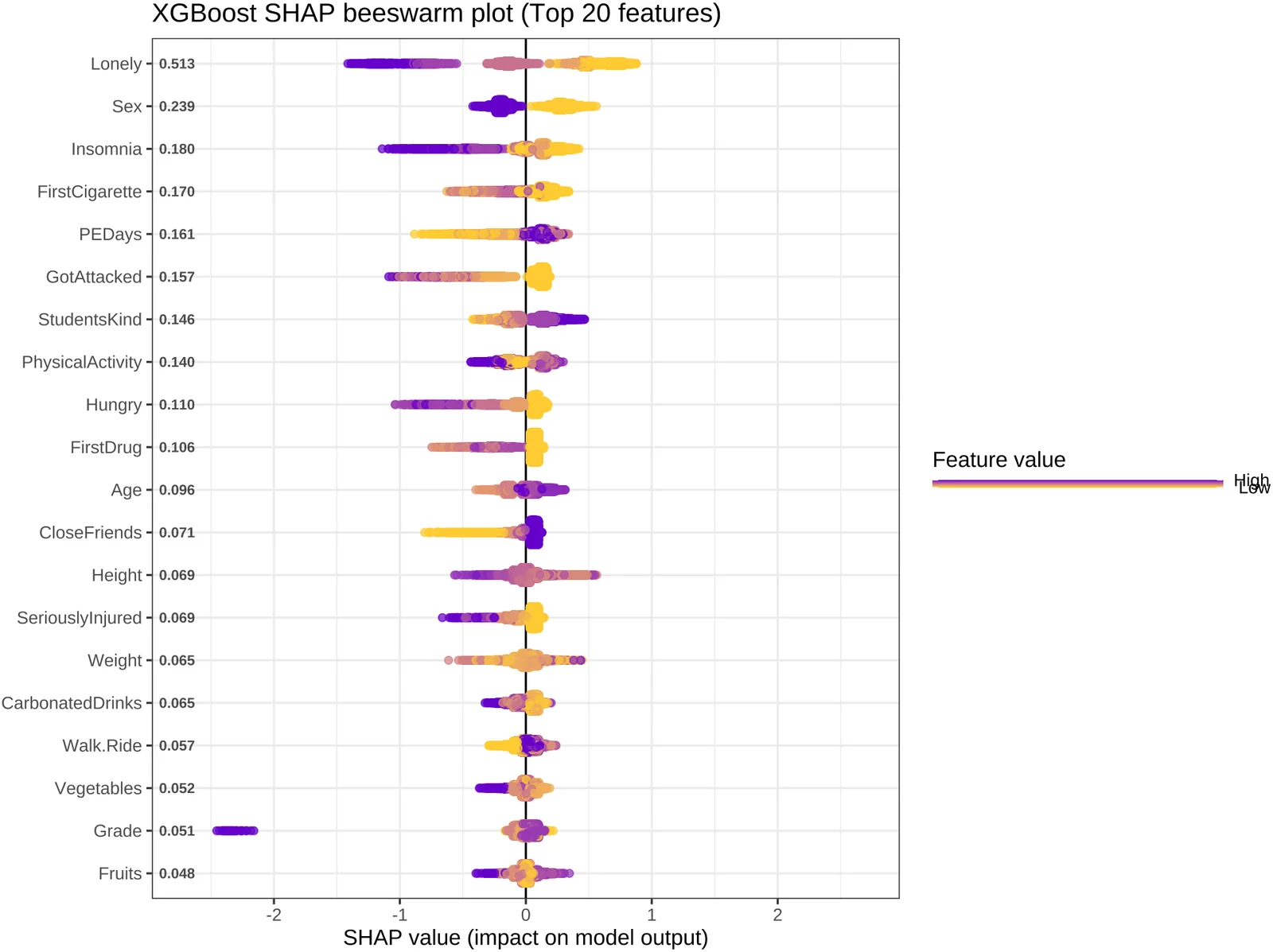
Global SHAP beeswarm plot showing the contribution and distribution of the top 20 predictors in the XGBoost model. SHAP values represent feature contributions to the model output; positive SHAP values indicate contributions toward a higher predicted probability of suicide attempt after reversing the original class coding direction.

The definitions in the GSHS codebook support this interpretation. The suicide-attempt item assessed how many times students actually attempted suicide during the past 12 months. Loneliness and insomnia-related worry were also measured over the past 12 months. Physical activity was assessed as the number of days during the past 7 days on which students were physically active for at least 60 minutes per day. Walking or riding to school was assessed over the past 7 days. Physical education participation was measured as the number of days per week that students attended PE class during the school year. School peer climate was assessed by how often most students in school were kind and helpful during the past 30 days. Thus, the model combined emotional distress, sleep-related anxiety, school social climate, and structured or unstructured physical activity indicators across complementary time windows.

Early cigarette use was also among the leading SHAP-ranked predictors. This finding is important because tobacco-related behavior may represent more than a single substance-use exposure. It may reflect impulsivity, peer environment, emotional dysregulation, family context, or clustering with other adolescent health-risk behaviors. Longitudinal evidence has shown that early-onset tobacco use is associated with later suicide-related behavior, and meta-analytic evidence has linked smoking with suicidal ideation, suicide plan, suicide attempt, and suicide death (8, 11). In our model, early cigarette use should therefore be interpreted as part of a broader behavioral vulnerability profile rather than as an isolated causal factor.

The presence of physical attack and serious injury among the important predictors further supports the interpretation that suicide-attempt risk is embedded in interpersonal and environmental adversity. Adolescents who experience physical attack or recurrent injury may have greater exposure to violence, unsafe environments, impulsive behavior, or social conflict. Previous studies have documented co-occurrence between physical fighting, violence exposure, and suicide attempts among adolescents (15). In the present model, such variables may help identify adolescents whose risk is not purely internalizing, but also linked to external stressors and unsafe school or community contexts.

The role of school peer climate also deserves attention. In the baseline table, adolescents with suicide attempt were more likely to report that students in their school were never or rarely kind and helpful. This variable was also retained among the top SHAP-ranked predictors, suggesting that suicide-attempt risk may be closely related to perceived school connectedness and peer support. Prior studies have shown that stronger school connectedness is associated with lower adolescent suicidality, and longitudinal evidence has also linked social connectedness with lower suicidal thoughts and behaviors (7, 9). These findings are important for interpreting the physical activity results, because PE classes and school-based activity programs may influence mental health not only through exercise itself, but also through peer interaction, belonging, adult supervision, and school engagement.

A major contribution of this study is that physical activity and PE-related variables remained informative even after stronger psychosocial predictors were included. In the baseline table, adolescents with suicide attempt were more likely to report zero days of physical activity during the past week than those without suicide attempt, with proportions of 19.3% and 13.5%, respectively. They were also more likely to report no weekly PE attendance, with proportions of 15.3% and 9.7%, respectively. These findings suggest that physical inactivity and limited exposure to structured school-based movement are not neutral background characteristics. Rather, they may be part of a broader risk profile involving social withdrawal, sleep disturbance, weak school engagement, poorer peer support, and reduced participation in health-promoting routines. However, given that the cross-sectional design, these findings should not be interpreted as evidence that increasing PE participation would directly reduce suicide-attempt risk. Reduced physical activity or PE participation may represent downstream manifestations of psychosocial vulnerability, including loneliness, insomnia, depressive symptoms, social withdrawal, poor school engagement, or negative peer experiences. Therefore, longitudinal studies and intervention trials are required to determine whether improving physical activity opportunities can causally influence adolescent suicidal outcomes (**Figure 1**).

The SHAP and PDP analyses further suggest that the association between physical activity-related variables and predicted suicide-attempt risk was nonlinear rather than strictly dose-dependent. For PhysicalActivity, very low activity, particularly zero days with at least 60 minutes of physical activity during the past week, was associated with a higher predicted probability of suicide attempt after correcting for outcome coding. For PEDays, low PE participation similarly showed a contribution toward a higher predicted probability of suicide attempt. However, neither variable demonstrated a simple linear pattern in which every additional day uniformly reduced risk. This point is important. The results do not imply that more exercise automatically prevents suicide attempts. Instead, they suggest that insufficient physical activity and limited PE participation are actionable markers within a broader psychosocial risk profile.

This interpretation is supported by studies specifically examining movement behaviors and suicidality. A global analysis of adolescents from 48 countries reported sex-specific associations between physical activity and suicide attempts (6). Another study examining suicidal ideation and attempts in relation to physical activity emphasized that physical activity should be considered together with other adolescent health-risk behaviors (10). Evidence from 24-hour movement behavior research also suggests that adherence to movement guidelines may be associated with lower odds of suicidality in some adolescent subgroups (16). These findings are consistent with our SHAP and PDP results: physical activity is relevant to risk prediction, but its meaning is likely context-dependent and should not be reduced to a simple linear exposure.

The mental-health relevance of physical activity in adolescence is also biologically and psychosocially plausible. Physical activity may improve sleep regulation, stress reactivity, emotional control, self-efficacy, self-esteem, and body image. A review of reviews concluded that physical activity may have beneficial effects on mental health in children and adolescents, although the evidence varies by outcome and study design (3). A systematic review and meta-analysis also found that physical activity interventions may help reduce depressive symptoms in children and adolescents. These findings are relevant because depressive symptoms, anxiety-related sleep disturbance, and perceived social disconnection are central to many suicide-risk pathways.

The social context of physical activity may be particularly important. In our model, loneliness was the strongest predictor, and school peer kindness was also among the top SHAP-ranked features. This suggests that the potential value of PE and school-based activity may extend beyond exercise volume. PE classes, team-based games, active commuting initiatives, walking groups, and after-school activity clubs can create repeated opportunities for peer contact, adult supervision, belonging, and routine. A systematic review of sport participation in children and adolescents reported psychological and social benefits, including improved self-esteem, social interaction, and fewer depressive symptoms (5). Therefore, the most promising form of physical activity intervention for psychologically vulnerable adolescents may be socially supportive movement rather than competitive or performance-oriented exercise.

The local SHAP explanation illustrates how model interpretation can move from population-level ranking to individualized attribution. In the selected validation-set observation, the model produced a predicted probability of 0.9914 for the non-attempt class. After correcting for outcome coding, this local explanation indicates that the observed levels of loneliness, school peer kindness, sex, insomnia, PE days, physical activity, age, height, hunger, and first cigarette use collectively shifted prediction away from suicide-attempt risk (**Figure 2**). This figure demonstrates the practical value of explainable machine learning: it converts a numerical prediction into an interpretable profile that can be understood in terms of emotional distress, sleep, school climate, health-risk behavior, and movement-related factors. In school health practice, such individualized explanations may help identify which domains require attention for a given student.

From an implementation perspective, physical activity promotion is attractive because it is comparatively inexpensive, scalable, and acceptable when designed appropriately. Specialist mental-health resources are often limited in school settings, particularly in resource-constrained regions. In contrast, PE classes, playgrounds, walking or cycling routes, classroom activity breaks, and after-school activity clubs can often be implemented or strengthened using existing school infrastructure. Importantly, adolescents’ own preferences matter. A study of adolescent physical activity preferences found that most adolescents wanted to increase at least one type of physical activity, and many preferred doing activity with friends rather than family (4). This supports the idea that school-based activity programs should emphasize choice, peer participation, and social acceptability rather than compulsory or punitive exercise.

Economic evidence also supports the feasibility of using physical activity promotion as a public health strategy. An economic analysis of physical activity interventions reported that school-based physical activity interventions targeting children and adolescents ranked favorably in terms of cost per physical activity outcome (17). This does not mean that all school-based programs are automatically effective or cost-effective; rather, it suggests that the school setting is a realistic platform for low-cost population-level action when programs are well designed and implemented. In the context of our findings, physical activity promotion should complement, not replace, mental-health screening, counseling, crisis referral, sleep-health education, and tobacco-risk prevention.

The role of school peer climate deserves special attention. In the baseline table, adolescents with suicide attempt were more likely to report that students in their school were never or rarely kind and helpful. This variable also appeared among the top SHAP-ranked predictors. Evidence from school connectedness research indicates that higher school connectedness is associated with lower suicidal ideation and, in some groups, lower suicide-attempt risk (7, 8). This has direct implications for PE. A PE class that is competitive, exclusionary, or associated with embarrassment may not improve mental health and may even increase distress for vulnerable students. By contrast, inclusive and socially supportive PE may increase movement while also strengthening belonging, peer contact, and school connectedness. This may help explain why the SHAP and PDP patterns for activity-related variables were nonlinear.

These findings also align with an integrated mind–body perspective of adolescent mental health. Physical activity engagement is influenced not only by physiological capacity but also by psychological distress, self-perception, interpersonal experiences, and social context. In vulnerable adolescents, physical education may become a source of distress when associated with physical humiliation, body shame, peer exclusion, or excessive performance pressure. Therefore, exercise should not be considered universally protective; rather, its psychological effects depend on whether the activity environment promotes safety, belonging, and positive self-perception.

Recent evidence from exercise-based rehabilitation settings has demonstrated that psychological distress can negatively influence physical performance, supporting the bidirectional interaction between emotional and physical functioning (18). Broader theoretical perspectives have further emphasized the importance of considering mind–body integration in health promotion and clinical care (19).

The cumulative AUC analysis further supports the feasibility of a compact school-based risk profile. Predictors were ranked by SHAP importance and sequentially added to evaluate cumulative cross-validated AUC. The top 18 SHAP-ranked predictors achieved a cumulative cross-validated AUC of approximately 78.65%, while the final model achieved an independent validation AUC of approximately 79.28% (**Figure 3**). This suggests that much of the predictive signal may be retained using a relatively limited set of school-available variables. In real-world school health practice, a concise screening profile is more feasible than a complex system requiring extensive clinical assessment. Such a profile could include loneliness, sex, insomnia, early cigarette use, PE days, physical attack, school peer climate, physical activity, close friends, and injury-related indicators.

**Figure 3:**
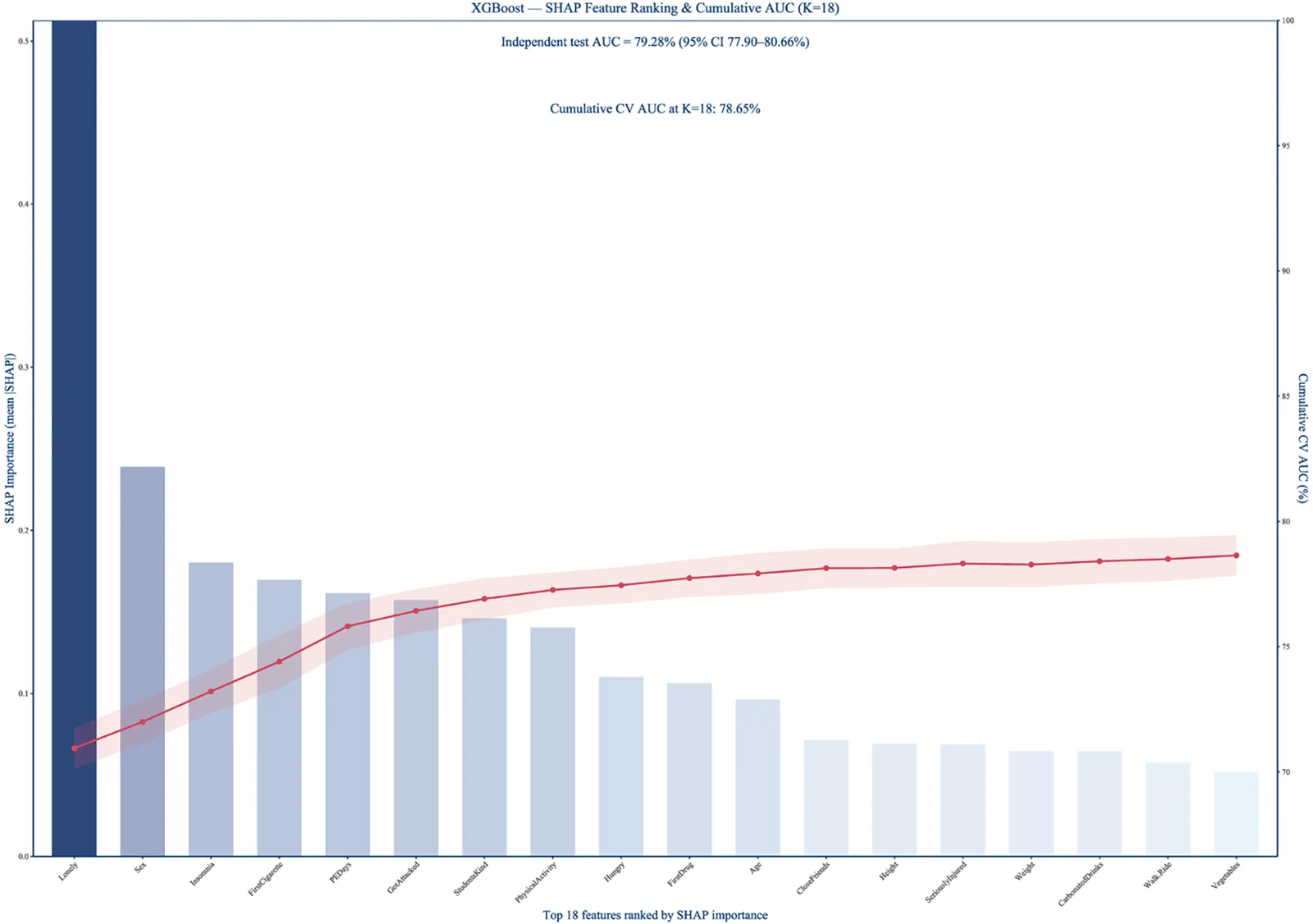
SHAP-based feature ranking and cumulative 10-fold cross-validated AUC for the top 18 predictors.

The intervention implication is therefore risk-stratified rather than purely universal. Adolescents characterized by loneliness, insomnia, weak peer connectedness, early cigarette use, low physical activity, and limited PE participation may represent a priority group for early support. For these students, the most promising intervention is unlikely to be high-intensity, competitive, or performance-oriented exercise. Instead, schools should consider inclusive, socially supportive physical activity programs that emphasize participation, safety, peer belonging, and routine movement. Examples include low-pressure team activities, supervised walking groups, active classroom breaks, after-school activity clubs, and PE sessions adapted for students with low confidence, poor sleep, or social isolation. In this framework, physical activity is not only an exercise exposure, but also a social, behavioral, and school-engagement intervention platform.

The explainable machine-learning approach adds value by linking prediction to prevention. Traditional regression models can estimate average associations, but may be less effective at communicating nonlinear relationships or individualized risk profile. SHAP analysis allowed us to rank predictors and interpret how each feature contributed to prediction, while PDPs helped visualize how predicted risk changed across selected predictor levels. Together, these methods suggested a practical prevention framework: loneliness and insomnia define the psychosocial core of risk, whereas identify potentially modifiable school-based indicators for targeted support. Beyond psychosocial and behavioral vulnerability, emerging evidence (2) suggests that biological pathways, including inflammatory alterations, may contribute to adolescent mental-health vulnerability. Inflammatory markers have been proposed as potential biological correlates of psychological distress and suicidal-related outcomes. Future predictive models may benefit from integrating psychosocial features with biological indicators to develop multidimensional risk-stratification frameworks.

Several limitations should be acknowledged. First, this was a cross-sectional study; therefore, causal relationships cannot be inferred. Physical inactivity may contribute to psychological vulnerability, but adolescents with loneliness, insomnia, or emotional distress may also be less likely to participate in physical activity or may experience PE negatively. Second, all variables were self-reported, which may introduce recall bias or social desirability bias, especially for sensitive variables such as suicide attempt, cigarette use, and sexual behavior. Third, no missing-data imputation was performed, and complete-case analysis may introduce bias if missingness was related to mental-health status or behavioral risk. Fourth, although the model was externally validated using Anguilla and Jamaica data, the training and validation datasets came from different countries and survey years, and cultural, educational, or policy differences may influence model transportability.

Another limitation is that GSHS measures physical activity and PE mainly by frequency rather than by intensity, enjoyment, quality, motivation, peer interaction, or inclusiveness. These unmeasured dimensions may be crucial for understanding why activity-related effects were nonlinear. A student may attend PE frequently but still experience body-image stress, peer exclusion, or excessive competition. Conversely, even moderate activity may be beneficial if it occurs in a supportive environment that strengthens peer connection and school belonging. Future longitudinal and intervention studies should distinguish between the quantity of physical activity and the psychosocial quality of the activity setting, and should test whether socially supportive PE programs can reduce loneliness, improve sleep, and lower suicide-related risk.

In conclusion, this explainable machine-learning study identified loneliness, sex, insomnia, early cigarette use, PE participation, physical attack, school peer climate, and physical activity as important contributors to adolescent suicide-attempt prediction. Loneliness and insomnia appear to define the psychosocial core of risk, whereas insufficient physical activity and low PE participation identify a modifiable, low-cost, school-based intervention window. These findings support the integration of school-based risk stratification with inclusive, socially supportive physical activity programs as part of broader adolescent mental-health promotion and suicide-prevention strategies.

## 5 Conclusion

In this cross-national analysis of GSHS data from Argentina, Anguilla, and Jamaica, an explainable XGBoost model showed acceptable performance for identifying adolescent suicide-attempt status, with an independent validation AUC of 0.7928. SHAP interpretation revealed that suicide-attempt risk was driven primarily by psychosocial vulnerability, especially loneliness and insomnia, while sex, early cigarette use, physical attack, school peer climate, physical activity, and PE participation also contributed meaningfully to prediction.

The key translational message is that loneliness and insomnia identify the core psychological vulnerability, whereas physical inactivity and limited PE participation represent potentially informative school-based indicators that may assist risk stratification and targeted support. Although the cross-sectional design precludes causal inference, these findings suggest that school-based suicide-prevention strategies should not stop at risk screening. They should integrate early psychological support with inclusive, socially supportive physical activity and PE programs that strengthen peer connection, school belonging, sleep health, and routine movement.

In short, explainable machine learning identified not only who may be vulnerable, but also where schools may intervene.

### 5.1 Limitations

Several limitations should be noted. First, this was a cross-sectional study, so temporal sequence and causality cannot be inferred. Second, all variables were self-reported, and GSHS measured physical activity and PE participation mainly by frequency, without information on activity intensity, PE quality, enjoyment, competitiveness, peer interaction, or school-level intervention context. Therefore, physical activity and PE should be interpreted as modifiable school-based indicators rather than proven causal protective factors. Finally, although the model was validated in different countries, survey year, culture, and school systems may affect model transportability. Further longitudinal and intervention studies are needed to confirm whether socially supportive PE and physical activity programs can reduce adolescent suicide-attempt risk.

## Data Availability

The original contributions presented in the study are included in the article/supplementary material. Further inquiries can be directed to the corresponding author/s.
